# Antimicrobial residues, non-typhoidal *Salmonella*, *Vibrio* spp. and associated microbiological hazards in retail shrimps purchased in Ho Chi Minh city (Vietnam)

**DOI:** 10.1016/j.foodcont.2019.106756

**Published:** 2020-01

**Authors:** Nguyen Thi Phuong Yen, Nguyen Thi Nhung, Nguyen Thi Bich Van, Nguyen Van Cuong, Le Tran Tien Chau, Huynh Ngoc Trinh, Chu Van Tuat, Nguyen Dong Tu, Nguyen Phu Huong Lan, James Campbell, Guy Thwaites, Stephen Baker, Juan Carrique-Mas

**Affiliations:** a Oxford University Clinical Research Unit, 764 Vo Van Kiet, Ho Chi Minh City, Viet Nam; b National Centre for Veterinary Hygiene Inspection No. I, 28 Ngo 78, Giai Phong, Ha Noi, Viet Nam; c National Institute of Hygiene and Epidemiology, 1 Yersin, Hanoi, Viet Nam; d Hospital of Tropical Diseases, 764 Vo Van Kiet, Ho Chi Minh City, Viet Nam; e Centre for Tropical Medicine and Global Health, Nuffield Department of Clinical Medicine, Oxford University, Old Road Campus, Oxford, OX3 7FZ, United Kingdom

**Keywords:** Shrimp, Non-typhoidal *Salmonella*, *Vibrio*, Residues, Antimicrobial resistance, Vietnam

## Abstract

We investigated antimicrobial residues, non-typhoidal *Salmonella* (NTS), *Vibrio* spp. and their associated antimicrobial resistance (AMR), in shrimps locally purchased in Ho Chi Minh City (Vietnam). In addition, we investigated the relationship between AMR in NTS, *Vibrio* spp. and antimicrobial residue in the same sample. A total of 40 samples of shrimp heads/shells from different retail sources was cultured using ISO 6579–1:2017 (NTS) and ISO/TS 21872–1:2007 (*Vibrio* spp.). Phenotypic antimicrobial susceptibility was investigated using Vitek (NTS, 34 antimicrobials) and disk diffusion (*Vibrio* spp., 12 antimicrobials). A total of 9 (22.5%) samples contained antimicrobial residue, including tetracyclines, fluoroquinolones, sulfonamides and macrolides (in 7.5%, 7.5%, 2.5% and 2.5% of samples, respectively). Shrimp samples from supermarkets had a higher prevalence of antimicrobial residue than those purchased in street markets (50% vs. 13.3%) (*p* = 0.049). A total of 30 (75%) samples were contaminated with NTS. All samples contained *Vibrio* spp., with *V. parahaemolyticus* being most common (87.5% samples). A total of 58.9% NTS isolates were multidrug resistant. With regards to the highest priority, critically important antimicrobials, the highest resistance corresponded to quinolones (14.4–47.8%), followed by 3rd and 4th generation cephalosporins (3.3–7.8%). *Vibrio* spp. isolates were characterised by their high resistance against ampicillin (82.7%) and 3rd generation cephalosporins (8.3–16.5%). Extended Spectrum Beta-Lactamase (ESBL) activity was detected in 28.1% *V. parahaemolyticus* isolates. Half of ESBL-positive *V. parahaemolyticus* strains harboured *bla*_CTX-M1_. We found an association between the presence of residues and the number of resistances for NTS (p = 0.075) and *Vibrio* spp. isolates (p = 0.093) from the same sample. These findings suggest that the presence of residues may contribute to the selection of AMR in foodborne pathogens in shrimps. Authorities should strengthen policies aiming at restricting inappropriate antimicrobial usage in shrimp farming, and step up monitoring of antimicrobial residues and food-borne pathogens at retail in Vietnam.

## Highlights

• Highest prevalence of antimicrobial residues found in shrimps from supermarkets.
• 75% samples were contaminated with non-typhoidal *Salmonella* (NTS).
• Isolates from samples with antimicrobial residues had highest prevalence of phenotypic resistance.
• Extended Spectrum Beta-Lactamase activity detected in 28.1% *V. parahaemolyticus* isolates.

## 1 Introduction

Antimicrobial resistance (AMR) is one of the greatest threats to our society (O'Neill, 2016). Among other sources, humans may acquire AMR bacterial infections or AMR-encoding genes through the consumption of contaminated food, including fish and shellfish (Cabello et al., 2013, Likotrafiti et al., 2018). In recent years shrimp farming has rapidly increased, reaching a global production of 3.2 million metric tons in 2017, much of it taking place in Asia (Anon., 2018). This increase is happening in a context of rapid globalization of markets, as well as the threat of climate change (Lauria et al., 2018). Antimicrobials are widely used in shrimp and aquaculture production, both to treat and prevent diseases (Henriksson et al., 2018). Contamination of aquaculture food products with antimicrobial residues represents a potential health hazard to the consumer due to food poisoning, the development of allergy problems, changes of the intestinal flora, as well as the emergence and subsequent spread of antimicrobial resistance (Okocha, Olatoye, & Adedeji, 2018).

Non-typhoidal *Salmonella* (NTS) and certain *Vibrio* spp. are major microbiological hazards associated with shrimp and seafood consumption (Baker-Austin et al., 2018, Tusevljak et al., 2012). *Vibrio parahaemolyticus* is the leading cause of seafood-borne bacterial gastroenteritis in the world. Both its thermostable direct hemolysin (*tdh*) and tdh-related hemolysin (*trh*) are considered major virulence factors of this micro-organism (Raghunath, 2015). In the late 1990s’, *V. parahaemolyticus* was implicated in a large outbreak of enteric disease in central Vietnam, with 523 cases reported (Chowdhury et al., 2004).

NTS is a major cause of gastroenteritis worldwide (Majowicz et al., 2010). In Vietnam, NTS is recognised as a major cause of pediatric diarrhoea (Thompson et al., 2012). There is also evidence of an increase in the incidence of severe invasive infections in hospitalised patients associated due to this organism (Lan et al., 2016, Nga et al., 2012).

The Vietnamese shrimp industry has experienced a considerably expansion over recent years, with most of its production being aimed the export market (mostly to the USA, Europe and Japan). In 2017, shrimp exports made up about half of the total Vietnam seafood exports, with sales worth 3.8 billion US$ (Hong, Hien, Thu, & Lebailly, 2017).

Shrimp exports are regularly screened for their microbiological safety by the companies themselves. However, little is known about the microbiological safety of shrimps available for domestic consumption. Therefore, the aims of this study were: (1) to investigate major foodborne hazards associated with shrimps from local retail sites in Ho Chi Minh City (HCMC), Vietnam, such as antimicrobial residues, NTS and *Vibrio* spp.; and (2) to characterise the AMR profile of these organisms, including the presence of Extended Spectrum Beta-Lactamases (ESBL) and colistin resistance. In addition we investigated the relationship between the presence of AMR in the two bacterial species and antimicrobial residues in the same batches, which to our knowledge has not been previously investigated.

## 2 Methods

### 2.1 Sample collection and identification

Batches of shrimps (250–300 g each) were purchased from 40 different retail sites located in 10 districts of HCMC (Vietnam) from March to June 2018. In order to maximize the diversity of sources, from each district three street markets and one supermarket were selected. From each retail site, one batch of live or dead shrimps (chilled, not frozen) was purchased. Shrimps were collected into a clean plastic bag, and were transported to the laboratory within 2 h in an ice-containing box. Five representative specimens *per* batch were weighted using precision scales. Shrimp species were identified based on their morphological features. Using a pair of sterile scissors, the heads, legs and exoskeleton were separated from the muscle tissue, and were subsequently pooled (shell mix). Muscle tissue samples were investigated for the presence of antimicrobial residues, and the shell mixes were investigated for NTS and *Vibrio* spp.

### 2.2 Antimicrobial residue analyses

Shrimp muscle tissue samples were investigated for antimicrobial residues using a hierarchical approach. Firstly, they were screened using PremiTest (R-Biopharm AG, Germany), an assay based on the inhibition growth of *Bacillus stearothermophilus* spores. Positive or inconclusive result samples were then examined for the presence of macrolides, amphenicols, tetracyclines, β-lactams and sulfonamides antimicrobial classes, as well as for the presence of chloramphenicol, streptomycin and gentamicin/neomycin using a Charm II analyzer 7600 (Charm Sciences, USA) (Gaudin, Juhel-Gaugain, Moretain, & Sanders, 2008). Samples that tested positive by Charm II were then confirmed for specific antimicrobials within each class by Ultra-High Performance Liquid Chromatography Tandem Mass Spectrometry (LC-MS/MS). In addition, PremiTest-positive samples were investigated for quinolones by LC-MS/MS. (See Table A with the list of antimicrobials investigated by LC-MS/MS).

### 2.3 Isolation of NTS and vibrio spp.

The shrimp shell mixes were investigated for NTS using a modified ISO 6579–1:2017 method. Briefly, from each sample 25 g of homogenized shell mix was pre-enriched in 225 mL buffered peptone water (BPW, Oxoid, UK) at 37 °C for 18 h. A loop of pre-enrichment media was then inoculated on Modified Semi-solid Rappaport-Vassiliadis (MSRV, Oxoid, UK), and incubated at 41.5 °C for 24 h. and positive growth was further inoculated on chromogenic Rambach agar (CHROMagar, France) and incubated at 37 °C for 24 h (Carrique-Mas, Barnes, McLaren, & Davies, 2009). Matrix-Assisted Laser Desorption Ionization Time-of-Flight Mass Spectrometry (MALDI-TOF MS) (Bruker, Germany) was used to investigate the species identity of three suspected (pink) isolates from each culture. NTS isolates were further classified as either group B, C, D, E or ‘others’ according to the Kauffmann-White scheme using relevant poly-O antiserum (Grimont P.A. & Weill, 2007). Shrimp shell mixes (25 g) were also investigated for the presence of *Vibrio* spp. using a modification of the ISO/TS 21872–1:2007 method. Briefly, the steps were: (1) 25 g of the shell mix was suspended in 225 mL of alkaline saline peptone water (ASPW) at 41.5 °C for 24 h; (2) a loop of enrichment was cultured on thiosulfate citrate bile and sucrose agar (TCBS, Oxoid, UK) at 37 °C for 24 h. Four suspected *Vibrio* spp. isolates from each sample were confirmed by MALDI-TOF.

### 2.4 Antimicrobial susceptibility testing

All confirmed NTS isolates were tested for their antimicrobial susceptibility against a panel of 34 antimicrobials belonging to 11 classes by Vitek (bioMérieux, Marcy l’Etoile, France) (Livermorea et al., 2002) (33 antimicrobials), as well as by Etest (BioMérieux, France) (colistin). All *Vibrio* spp. isolates were tested using the disk diffusion method for 12 antimicrobials representative of eight classes (Oxoid, UK). The full list of antimicrobials investigated is displayed in Tables C4 and C5. NTS and *Vibrio* spp. isolates were classified as susceptible, intermediate or resistant according to CLSI guidelines (M100-S27 for NTS, M45-A2 for *Vibrio* spp.) (Anon, 2010, Anon, 2017a). A strain was defined as ‘multidrug resistant’ (MDR) if it was fully resistant to antimicrobials belonging to at least three different classes. The potential production of ESBLs was investigated by the ‘comparative disk diffusion method’, using cefotaxime and ceftazidime disks alone, as well as in combination with clavulanate (Anon., 2017a). Antimicrobial susceptibility results were sorted according to the WHO list of antimicrobials of human health importance (Anon., 2017b).

### 2.5 Determination of serovar identity of NTS

NTS isolates were further classified as belonging to either group B, C, D, E or ‘others’ according to the Kauffmann-White scheme using poly-O antiserum (Grimont P.A. & Weill, 2007). From each sample, one isolate representative of each a serogroup-antimicrobial susceptibility testing result pattern was investigated by Multi-Locus Sequence Typing (MLST). The MLST scheme followed is based on seven loci *aro*C, *dna*N, *hem*D, *his*D, *pur*E, *suc*A and *thr*A (Yun et al., 2015).

### 2.6 Investigation of tdh and trh genes of vibrio spp. by PCR

The presence of genes encoding a thermostable direct hemolysin (*tdh*) and a tdh-related hemolysin gene (*trh*) was investigated by PCR in all *Vibrio* spp. isolates (Tada et al., 1992). Positive and negative control isolates were used. The positive control isolates originated from confirmed human cases.

### 2.7 Investigation of ESBL and plasmid mediated colistin resistance-encoding genes by PCR

The presence of *bla*_CTX-M(1, 2, 8, 9 and 25)_, *bla*_TEM_, *bla*_SHV_, and *bla*_OXA_ genes (all encoding extended-spectrum *β*-lactamases) was investigated by multiplex PCR (Dallenne, Da Costa, Decre, Favier, & Arlet, 2010) in all NTS and *Vibrio* spp. isolates that tested positive phenotypically for ESBL. The presence of plasmid-mediated genes (*mcr*-1 to *mcr*-5) among phenotypic colistin-resistant isolates was investigated by multiplex PCR (Rebelo et al., 2018).

### 2.8 Statistical analyses

We investigated 40 shrimp batches with the aim of determining the prevalence of residues, based on an expected prevalence of 8%, a 95% level of confidence and an 8% relative precision. We expected to obtain 50 NTS isolates from these batches. This sample size (50) allowed determining a prevalence of MDR of ∼27% (based on published data) with a 95% level of confidence and a 9% relative precision. The prevalence of contamination and resistance across variables was compared using chi-square tests. The level of agreement between presence of residues and presence of intermediate (or resistant) isolates in each shrimp batch sample was investigated using the kappa statistic.

## 3 Results

### 3.1 Shrimp samples

The 40 batches investigated included specimens of five shrimp species: white leg shrimp (*Litopenaeus vannamei*) (30) (22.5 g weight; 16.2 ± Standard Deviation (SD) 1.2 cm length), giant tiger shrimp (*Penaeus monodon*) (5) (22.0 g; 15.4 ± SD 1.4 cm), banana shrimp (*Penaeus merguiensis*) (3) (17.3 g, 12.5 ± SD 1.6 cm), greasy-back shrimp (*Metapenaeus ensis)* (1) (11.5 g; 11.5 ± SD 1.4 cm), and giant prawn (*Macrobrachium rosenbergii*) (34.3 g; 16.6 ± SD 1.1 cm). The descriptive data for the samples investigated, and the prevalence of PremiTest, NTS and *Vibrio* spp. sample positivity results are shown in Table 1.

**Table 1:** Description, prevalence of residues, NTS and *Vibrio* spp. among 40 shrimp batches purchased in HCMC.

Variable	No. samples	No. (%) positive							
		PremiTest	NTS (%)	*Vibrio* species					
				*V. parahaemolyticus*	*V. navarrensis*	*V. alginolyticus*	*V. cholerae non-O1*	*V. vulnificus*	*V. fluvialis*
*Type of retail site*									
Supermarket	10	5 (50.0%)	6 (60.0%)	9 (90.0%)	7 (70.0%)	4 (40%)	6 (60.0%)	2 (20.0%)	0 (0%)
Street market	30	4 (13.3%)	24 (80.0%)	26 (86.7%)	17 (56.7%)	17 (56.7%)	9 (30.0%)	7 (23.3%)	4 (13.3%)
*Shrimp species*									
White leg shrimp	30	8 (26.7%)	22 (73.3%)	26 (86.7%)	15 (50.0%)	16 (53.3%)	12 (40.0%)	7 (23.3%)	4 (13.3%)
Giant tiger shrimp	5	1 (20.0%)	3 (60.0%)	4 (80.0%)	5 (100%)	2 (40.0%)	2 (40.0%)	1 (20.0%)	0 (0%)
Other species	5	0 (0%)	5 (100%)	5 (100%)	4 (80%)	3 (60%)	1 (20%)	1 (20%)	0 (0%)
*Condition*									
Alive	17	3 (17.6%)	12 (70.6%)	16 (94.1%)	8 (47.1%)	11 (64.7%)	4 (23.5%)	5 (29.4%)	4 (23.5%)
Dead	23	6 (26.1%)	18 (78.3%)	19 (82.6%)	16 (69.6%)	10 (43.5%)	11 (47.8%)	4 (17.4%)	0 (0%)
*Retail price (per kg)*									
≤170 k VND^a^	22	6 (27.3%)	14 (63.6%)	18 (81.8%)	14 (63.6%)	12 (54.5%)	9 (40.9%)	5 (22.7%)	1 (4.5%)
>170 k VND	18	3 (16.7%)	16 (88.9%)	17 (94.4%)	10 (55.6%)	9 (50%)	6 (33.3%)	4 (22.2%)	3 (16.7%)
*Total*	40	9 (22.5%)	30 (75.0%)	35 (87.5%)	24 (60.0%)	21 (52.5%)	15 (37.5%)	9 (22.5%)	4 (10.0%)

a VND=Vietnam Dong (1USD = 23 kVND).

### 3.2 Antimicrobial residues

Antimicrobial residues were detected in 9/40 (22.5%) samples by PremiTest. Shrimp samples from supermarkets had a higher prevalence of antimicrobial residues than those purchased in street markets (50% vs. 13.3%) (*χ*^2^ = 3.871, *p* = 0.049). Four of the nine PremiTest-positive samples were positive by Charm II, whereas the remaining five tested negative (Table 2). Tetracyclines, sulfonamides, and macrolides were detected by Charm II in 7.5%, 2.5%, and 2.5% of samples, respectively. Antimicrobials identified by LC-MS/MS included two tetracycline antimicrobials (tetracycline and oxytetracycline) and two fluoroquinolone antimicrobials (ciprofloxacin and flumequine). The Charm II macrolide-positive sample tested negative for both tylosin and erythromycin by LC-MS/MS. Two samples contained antimicrobial residues above the maximum residue limits (MRL) according to Vietnamese regulations (one contained tetracycline and sulfamethoxazole; another oxytetracycline, and ciprofloxacin). Oxytetracycline and flumequine were also found in two other samples although at concentrations below the MRL (43.7* μ*g/kg and 41.2* μ*g/kg, respectively).

**Table 2:** Results of antimicrobial residue testing by Charm II and LC-MS/MS among 9 shrimp samples that tested positive by PremiTest.

Sample ID	Description	Charm II (antimicrobial class)	LC-MS/MS (antimicrobial)	Concentration of antimicrobial active ingredient (*μ*g/kg)	MRL (*μ*g/kg)
1	White leg shrimp, dead	Tetracyclines	Tetracycline	590.7	100
		Sulfonamides	Sulfamethoxazole	157.6	100
			Flumequine^a^	38.5	200
2	White leg shrimp, dead	Tetracyclines	Oxytetracycline	122.2	100
			Ciprofloxacin*	30	Not allowed
3	White leg shrimp, dead	Macrolides	ND	–	–
			Flumequine^a^	41.2	200
4	White leg shrimp, dead	Tetracyclines	Oxytetracycline	43.7	100
5	White leg shrimp, dead	ND	ND	–	–
6	White leg shrimp, live	ND	ND	–	–
7	White leg shrimp, live	ND	ND	–	–
8	White leg shrimp, live	ND	ND	–	–
9	Tiger shrimp, dead	ND	ND	–	–

a Highest priority, critically important antimicrobial; ND=Not detected; MRL = Maximum Residue Limits according to Vietnamese regulation.

### 3.3 Prevalence of contamination with NTS serovars and vibrio spp.

A total of 90 NTS isolates were recovered from 30/40 (75%) shrimp batches (Table 1). The prevalence of NTS among samples purchased in street markets was higher than that from supermarket samples, although this difference was not significant (80% vs. 60%; *χ*^2^ = 0.711; p = 0.399). There was lower probability of recovering NTS from PremiTest-positive samples than from PremiTest-negative samples (5/9 vs. 25/31), although this difference was not significant (Fisher’s exact test, p = 0.190). MLST was performed on 62 isolates with a unique serogroup-antimicrobial susceptibility pattern. The remaining 28 isolates were assigned to serovar based on MLST results of isolates recovered from the same sample and with the same serogroup-AST pattern. A total of 28 MLST sequence types (ST) corresponding to 25 NTS serovars were identified. One NTS strain (Group B) could not be assigned to ST, and therefore its serovar identity was not determined (Table B). The most prevalent serovars identified were Braenderup (present in 20% samples), Anatum (16.7% samples), Saintpaul (13.3% samples), Rissen and Litchfield (10% samples each). All (100%) samples were positive for *Vibrio* species, yielding 133 isolates. Among six *Vibrio* species, *V. parahaemolyticus* was the most common species (87.5% samples), followed by *V. navarrensis* (60%), *V. alginolyticus* (52.5%), *V. cholerae non-O1* (37.5%), *V. vulnificus* (22.5%) and *V. fluvialis* (10%) (Table 1).

### 3.4 Antimicrobial susceptibility among NTSisolates

**Fig. 1:**
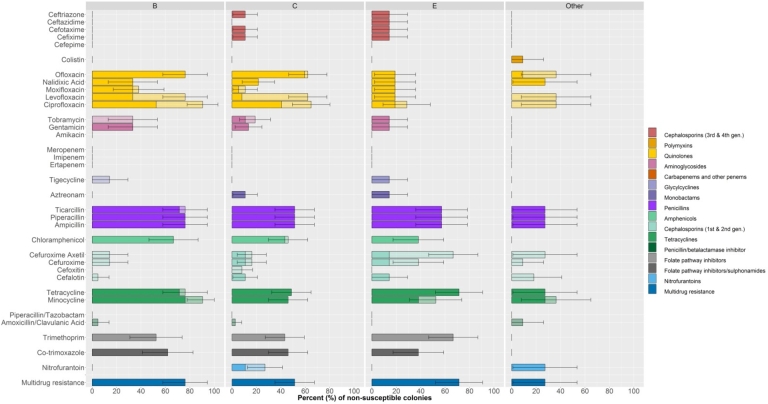
Phenotypic resistance of NTS isolates by group. Pale bars indicate the percent of isolates showing intermediate resistance; dark bars indicate percent of isolates with full resistance. 95% binomial confidence intervals have been drawn around the percentage of resistant plus intermediate resistant isolates.

Among highest priority-critically important antimicrobial classes, the highest prevalence of resistance corresponded to quinolones (nalidixic acid, ciprofloxacin, ofloxacin, levofloxacin, moxifloxacin) (range 14.4–47.8%), followed by 3rd and 4th generation cephalosporins (cefixime, cefotaxime, ceftazidime and ceftriazone) (3.3–7.8%). Among high priority-critically important antimicrobials, resistance was highest against aminoglycosides (16.7% gentamicin and 7.8% tobramycin), monobactams (7.8% aztreonam), and glycylcyclines (3.3% tigecycline) (Fig. 1, Table C). A total of 58.9% isolates were MDR. The highest prevalence of MDR corresponded to Group B isolates (76.2%; 95% CI 58.0–94.4%), followed by Group D (75.0; 95% CI 32.6–100%) and Group C (51.4%; 95% 35.2–67.5%). Seven isolates (7.8%) (from 3 samples) were identified as ESBL-positive. They were identified as serovars Infantis (3), Give (3) and Braenderup (1). The isolates identified as Infantis and Give (three of each, from two different samples) had identical antimicrobial susceptibility profile.

### 3.5 Antimicrobial resistance of vibrio spp. isolates

**Fig. 2:**
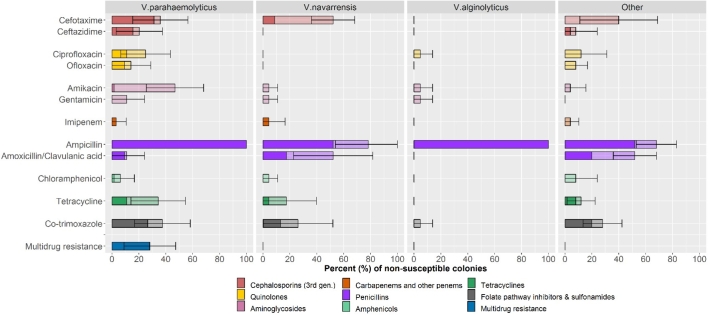
Phenotypic resistance of *Vibrio* spp. isolates. Pale bars indicate the percent of isolates showing intermediate resistance; dark bars indicate percent of isolates with full resistance. 95% binomial confidence intervals have been drawn around the percentage of resistant plus intermediate resistant isolates.

Results of antimicrobial susceptibility testing of 133 of *Vibrio* spp. against 12 antimicrobial drugs are shown in Fig. 2 and in Table D. The highest prevalence of resistance corresponded to ampicillin (82.7%), followed by co-trimoxazole (18.8%) and 3rd generation cephalosporins (16.5% cefotaxime; , 8.3% ceftazidime). All (100%) *V. parahemolyticus* and *V. alginolyticus* isolates were fully resistant to ampicillin. The prevalence of resistance against amoxicillin-clavulanic, penems, aminoglycosides, tetracyclines, quinolones and phenicols was <11.3% in all cases. A total of 18/64 (28.1%) *V. parahaemolyticus* were ESBL producers; however none (0%) of the 69 non-*V. parahaemolyticus* strains were ESBL producers. Overall, 18 of 133 (13.5%) *Vibrio* spp. isolates were MDR, but this percent was 28.1% among *V. parahemolyticus*, and 0% among other *Vibrio* species.

### 3.6 Detection of toxin-encoding genes

None of the 133 *Vibro* spp. isolates tested positive for either the *tdh* or *trh* genes.

### 3.7 Detection of ESBL genes in NTS and vibrio spp. isolates

The phenotypically ESBL-positive serovar Branderup isolate tested negative for all ESBL genes investigated. All three serovar Infantis isolates (from the same sample) were positive for *bla*_CTX-M9_. The three serovar Give isolates tested positive for both *bla*_CTX-M1_ and *bla*_TEM_. Interestingly, all isolates were fully susceptible to cefepime and cefoxitin. A total of 9/18 (50%) ESBL-positive *V. parahaemolyticus* strains were positive for *bla*_CTX-M1_. In addition, one of these isolates tested positive to the *bla*_TEM_ gene.

### 3.8 Relationship between residues, and AMR in NTS and vibrio spp. isolates

**Fig. 3:**
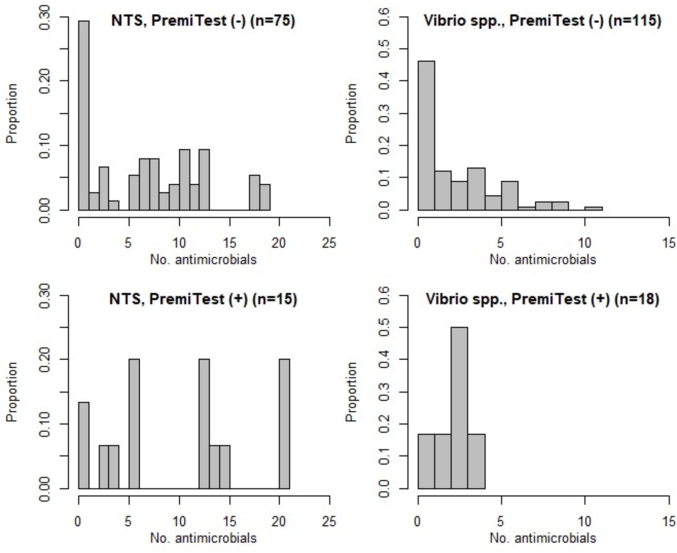
Number of phenotypic resistances among NTS and *Vibrio* spp. isolates from PremiTest-positive and PremiTest-negative shrimp samples.

NTS isolates from shrimp samples that tested positive to PremiTest (n = 15) were resistant to a median of 10 antimicrobials [IQR 3–13], whereas NTS isolates from samples testing negative (n = 75) were resistant to 5 antimicrobials [IQR 0–9] (Wilcoxon test W = 724.5, p = 0.075). *Vibrio* spp. isolates from PremiTest-positive samples (n = 29) were resistant to a median of 1 [IQR 1–4] antimicrobial, compared with 1 [IQR 1–2] among *Vibrio* spp. from PremiTest-negative samples (n = 104) (Wilcoxon = 1792; p = 0.093) (Fig. 3). We found fair agreement between presence of residue (PremiTest) and presence of co-trimoxazole and ciprofloxacin resistant NTS from the same sample (kappa values 0.265 and 0.365; p ≤ 0.016). We also found a fair agreement between presence of a quinolone residue and ciprofloxacin resistance in *Vibrio* spp. isolates from the same sample (kappa = 0.383; p = 0.005). In addition, there was a moderate agreement between samples that contained ESBL-positive *Vibrio* spp. and ESBL-positive NTS isolates (kappa = 0.515, p < 0.001) (Table D). However, in neither of the two samples that contained both phenotypically ESBL-positive NTS and *Vibrio* spp. we could demonstrate the presence of the same genes: in one sample *V. parahaemolyticus* was positive for both *bla*_TEM_ and *bla*_CTX-M1_, whereas no ESBL genes were detected in the NTS isolate; in the other, NTS harboured the *bla*_CTX-M9_, wheras *V. parahaemolyticus* tested negative for all ESBL genes.

## 4 Discussion

This study evidenced a high prevalence of contamination of shrimp samples with antimicrobial residues (22.5%), NTS (75%), and *Vibrio* spp. (100%). This result is in line with a previous survey of shrimps from local markets in the Red River and Mekong Delta regions of Vietnam (13.0% and 33.3%, respectively) (Pham et al., 2015), but higher than previous results reported from the Vietnamese provinces of HCMC, Thai Binh and Nha Trang (8.8, 1.8 and 3.2%, respectively) (Uchida et al., 2016). However, we were only able to establish the identity of the antimicrobial residue in 4 of 9 samples that tested positive by a bacterial inhibition test. This may be the result of a false-positive result in our screening test, or (more likely) due to the presence of antimicrobial residues not investigated in this study. All antimicrobials confirmed in our samples (tetracyclines, ciprofloxacin, flumequine) are known to be commonly used in Vietnamese aquaculture (Pham et al., 2015), although levels of flumequine (two samples) and oxytetracycline (one sample) where within the legal limits. We found considerable agreement between the screening results by Charm II and the residues detected by LC-MS/MS. The exception was a macrolide-positive sample by Charm that subsequently tested negative for both erythromycin and tylosin. We hypothesize that this may reflect the use of spiramycin, another macrolide antimicrobial known to be used in aquaculture (Sekkim & Kum, 2011) that was not investigated. It is likely that the observed prevalence of residues in shrimp samples reflects the use of antimicrobials late in the production cycle, thought to be common practice in Vietnamese aquaculture (Pham et al., 2015). A surprising finding was the higher prevalence of residues in shrimps procured from supermarkets than from (more informal) street markets. This suggests that intensive production systems (normally associated with the supermarket supply chains) may be associated with a higher levels of antimicrobial usage. This is consistent with a previous study that reported a higher prevalence of residues in shrimp products procured in supermarkets compared with wholesale markets (Uchida et al., 2016). The observed prevalence of contamination with NTS in this study (75%), is higher than in previous studies from Vietnam, China, Thailand and Bangladesh (prevalence levels ranging between 13% and 55%) (Minami et al., 2010, Phan et al., 2005, Pinu et al., 2007, Uddin et al., 2015). A recent study investigating NTS in 25 g meat samples purchased across markets in Vietnam resulted in a comparatively lower prevalence (71.8% in chicken, 70.7% in pork and 62.2% in beef samples) (Nhung et al., 2018). In contrast with the antimicrobial residue results, shrimp samples purchased in street markets had higher prevalence of NTS contamination than shrimps purchased in supermarkets, although this difference was not statistically significant. We hypothesize that this may reflect deficiencies in the cold chain associated with street markets. A study in Thailand reported absence of NTS in shrimps purchased in supermarkets, compared with a 50.2% prevalence among shrimps purchased in street markets (Minami et al., 2010).

We found a considerable serovar diversity (25) of NTS in our samples, with serovars Braenderup, Anatum, Saintpaul and Rissen accounting for 46.7% of all NTS isolates. Most serovars detected in shrimps have been isolated in outbreaks of human disease in different countries. A study from 2004 on 56 human clinical (diarrhoea, fever) in Vietnam identified 12 different serovars (Vo et al., 2006), of which four (Anatum, Albany, Typhimurium and Enteritidis) were detected in our shrimp samples. However, in that study, serovars Typhimurium and Enteritidis accounted for 50% of human cases, whereas in our study they only accounted for 6.6% of all isolates. A more recent study on invasive (bloodstream infection) isolates identified 19 serovars, of which 8 were identified in our study (Lan et al., 2016). Surprisingly, serovar Weltevreden, which was predominant in a previous study on shrimp farms in the Mekong Delta of Vietnam (Uddin et al., 2015) was found only in one sample, and was a pansusceptible strain. A possible explanation for this discrepancy is that NTS contamination of shrimps mainly occurs after harvesting. It has been shown experimentally that seafood is an excellent nutrient media supporting vigorous post-harvest NTS multiplication at ambient temperatures (Kumar, Datta, & Lalitha, 2015). Since no routine diagnostic or surveillance data exists for human salmonellosis, it is not possible to know to what extent shrimps are a significant source of NTS to the community in Vietnam. A total of 58.9% NTS isolates were MDR, a figure higher compared with overall MDR prevalence in isolates from meat in a previous study using the same antimicrobial panel (52.2%) (Nhung et al., 2018). Overall resistance to ciprofloxacin (33.3%) is of great concern, since this antimicrobial is often used to treat enteric infections. We found that 7.8% isolates were ESBL-producers and were fully resistant to at least one of the four 3rd-4th generation cephalosporins investigated. ESBL-producing NTS organisms were identified as belonging to serovars Braenderup (1), Infantis (3), and Give (3). These ESBL-producing isolates were MDR (including quinolone resistance), although were susceptible to carbapenems. The latter is the first choice drug in the treatment of ESBL-producing microorganisms (Zhanel et al., 2007).

All (100%) retail shrimp samples were contaminated with *Vibrio* spp., being *V. parahaemolyticus* the most prevalent species (87.5% samples). Other *Vibrio* species were isolated in 10.0–60.0% samples. These levels of contamination are comparable with studies in northern Vietnam (99.5% prevalence) (Tra et al., 2016) and Malaysia (100%) (Letchumanan, Yin, Lee, & Chan, 2015), although higher than results from Turkey (67%) (Mus, Cetinkaya, & Celik, 2014), confirming that *Vibrio* spp. organisms are omnipresent in the shrimp farm aquatic environment (Gopal et al., 2005). In addition to *V. parahaemolyticus*, both *V. vulnificus* and *V. cholerae* non-O1 are also known to cause severe human disease (Deshayes et al., 2015). We did not, however, find evidence of any of the two major virulence genes investigated (*tdh* and *trh*) in any of the 133 *Vibrio* spp. isolates. Previous research in Malaysia has shown a low prevalence of *tdh* (4%) and *trh* (12%) genes in non-clinical *V. parahaemolyticus* isolates (Paydar, Teh, & Thong, 2013). In a study on 47 environmental isolates from India, only 4.2% and 2.1% harboured the *tdh* and *trh* genes, respectively (Koralage et al., 2012). However there was no evidence of these genes in isolates investigated in northern Vietnam (Tra et al., 2016), Hong Kong (Wong, Liu, Wan, & Chen, 2012), or Sri Lanka (Koralage et al., 2012). In the late 1990s’, *V. parahaemolyticus* was implicated in a large outbreak of enteric disease in central Vietnam, with 548 cases reported (Tuyet et al., 2002). It was determined in further analyses that the prevailing serovar changed over time (O3:K6 in 1997, O4:K68 in 1998, O1:K25 in 1998–1999), and that 85% clinical isolates harboured either the *tdh* or *trh* genes (Chowdhury et al., 2004).

We found that 82.7% of *Vibrio* spp. isolates were resistant to ampicillin (100% for *V. parahaemolyticus* and *V. alginolyticus*)*.* This prevalence was comparable to published resistance levels among *V. parahaemolyticus* isolates from shrimps in northern Vietnam (87%) and Malaysia (82%) (Letchumanan et al., 2015, Tra et al., 2016). A total of 31.3% *V. parahaemolyticus* isolates were resistant to third generation cephalosporins and 28.1% were ESBL producers. In about half of those strains, the gene carried was *bla*_CTX-M1_. However, in another half the molecular basis for ESBL activity could not be established. Given that these genes are highly mobile, and are often inserted in plasmids and transposons, we hypothesize that *V. parahaemolyticus* may act as a reservoir of ESBL genes (Canton, Jose, & Galan, 2012). Interestingly we found an association between the presence of ESBL in NTS and *V. parahaemolyticus* isolated from the same sample.

The study confirmed the association between presence of antimicrobial residues and phenotypic resistance in NTS and *Vibrio* spp. in the same samples. This may be a reflection of AMR selection during the farming process, or may alternatively reflect post-harvesting contamination. The latter would be likely if the antimicrobial residue in the sample contributed to preferentially select for contamination with more resistant strains.

This study provides evidence of high levels of contamination with antimicrobial residues, NTS and *Vibrio* spp. among shrimps purchased in retail sites in HCMC. We found a high prevalence of MDR among NTS, with worryingly high levels of quinolone resistance. Although most *Vibrio* spp. isolates are unlikely to be pathogenic, the high carriage levels of ESBL in *V. parahaemolyticus* is of concern. We recommend authorities to enforce existing policies aiming at restricting inappropriate antimicrobial usage on shrimp farms, stepping up hygiene conditions during harvesting, transporting and retailing of shrimps, and to establish monitoring of antimicrobial residues, NTS and *Vibrio* spp. (focused on ESBL and virulence factors) in Vietnam. These findings should encourage the establishment of microbiological surveillance systems focused on health hazards in aquaculture food products, as well as strengthening laboratory capacity to enable comparisons between NTS and *Vibrio* spp. isolates from shrimps and human cases of disease.

## Conflicts of interest

The authors declare no conflict of interest.

## Figures and Tables

**Table A:** Panel of antimicrobial residues tested by LC-MS/MS.

WHO category	Antimicrobial class	Antimicrobial
Highest priority-critically important	Fluoroquinolones	Enrofloxacin, ciprofloxacin, norfloxacin, flumequine
	Macrolides	Tylosin, erythromycin
Highly important	Tetracyclines	Tetracycline, oxytetracycline, chlortetracycline
	Sulfonamides	Sulfadiazine, sulfamethazine, sulfaquinoxaline, sulfamethoxazole

**Table B:** Serovar identity of 62 NTS isolates investigated by MLST.

No.	Serogroup	Serovar	Sequence type (ST)	Number of isolates
1	B	Saintpaul	50	3
	B	Saintpaul	27	2
2	B	Derby	40	4
3	B	Paratyphi B	42	2
4	B	Typhimurium	36	2
5	B	Agona	13	1
6	B	Stanley	29	1
7	B	Unknown	Unknown	1
8	C	Braenderup	311	6
	C	Braenderup	22	3
9	C	Rissen	469	6
10	C	Bovismorbificans	1499	1
11	C	Infantis	32	1
12	C	Litchfield	214	3
13	C	Ohio	329	1
14	C	Albany	292	1
15	C	Bareilly	203	1
16	C	Kentucky	314	1
17	C	Potsdam	2039	1
18	D	Enteritidis	11	4
19	E	Anatum	64	8
20	E	Give	516	1
	E	Give	831	1
21	E	Senftenberg	14	1
22	E	Weltevreden	365	1
23	E	London	155	1
24	Other	Poona	1069	1
25	Other	Kedougou	1543	2
26	Other	Urbana	512	1

**Table C.1:** Phenotypic antimicrobial susceptibility results for 90 NTS isolates from shrimps, listed by antimicrobial class ranked by their importance according to the WHO classification. The figures correspond to the number of intermediate resistant, followed by the number of fully resistant isolates.

Class and antimicrobial	Group B (n = 21)								
	Agona	Derby	Paratyphi B	Saintpaul	Stanley	Typhimurium	Unknown	Intermediate resistant (%)	Fully resistant (%)
No. isolates	1	4	3	9	1	2	1		
Samples (n = 30) (%)	1 (3.3%)	2 (6.7%)	1 (3.3%)	5 (16.7%)	1 (3.3%)	2 (6.7%)	1 (3.3%)		
**Highest priority-critically important**									
Cephalosporins (3rd & 4th gen.)									
Cefepime	0│0	0│0	0│0	0│0	0│0	0│0	0│0	0(0%)	0(0%)
Cefixime	0│0	0│0	0│0	0│0	0│0	0│0	0│0	0(0%)	0(0%)
Cefotaxime	0│0	0│0	0│0	0│0	0│0	0│0	0│0	0(0%)	0(0%)
Ceftazidime	0│0	0│0	0│0	0│0	0│0	0│0	0│0	0(0%)	0(0%)
Ceftriazone	0│0	0│0	0│0	0│0	0│0	0│0	0│0	0(0%)	0(0%)
Polyymyxins									
Colistin	0│0	0│0	0│0	0│0	0│0	0│0	0│0	0(0%)	0(0%)
Quinolones									
Ciprofloxacin	1│0	4│0	2│0	0│9	0│1	1│0	0│1	8(38.1%)	11(52.4%)
Levofloxacin	1│0	4│0	0│0	3│6	1│0	0│0	0│1	9(42.9%)	7(33.3%)
Moxifloxacin	0│0	0│0	0│0	0│6	1│0	0│0	0│1	1(4.8%)	7(33.3%)
Nalidixic Acid	0│0	0│0	0│0	0│6	0│0	0│0	0│1	0 (0%)	7(33.3%)
Ofloxacin	0│1	0│4	0│0	0│9	0│1	0│0	0│1	0 (0%)	16(76.2%)
**High priority-critically important**									
Aminoglycosides									
Amikacin	0│0	0│0	0│0	0│0	0│0	0│0	0│0	0 (0%)	0 (0%)
Gentamicin	0│0	0│0	0│0	0│6	0│0	0│0	0│1	0 (0%)	7(33.3%)
Tobramycin	0│0	0│0	0│0	7│0	0│0	0│0	0│0	7(33.3%)	0 (0%)
Carbapenems and other penems									
Ertapenem	0│0	0│0	0│0	0│0	0│0	0│0	0│0	0(0%)	0(0%)
Imipenem	0│0	0│0	0│0	0│0	0│0	0│0	0│0	0(0%)	0(0%)
Meropenem	0│0	0│0	0│0	0│0	0│0	0│0	0│0	0(0%)	0(0%)
Glycylcyclines									
Tigecycline	0│0	0│0	0│0	3│0	0│0	0│0	0│0	3(14.3%)	0(0%)
Monobactams									
Aztreonam	0│0	0│0	0│0	0│0	0│0	0│0	0│0	0 (0%)	0 (0%)
Penicillins									
Ampicillin	0│1	0│4	0│0	0│9	0│1	0│0	0│1	0(0%)	16(76.2%)
Piperacillin	0│1	0│4	0│0	0│9	0│1	0│0	0│1	0 (0%)	16(76.2%)
Ticarcillin	0│1	0│4	0│0	0│9	0│1	0│0	1│0	1(4.8%)	15(71.4%)
**Highly important**									
Amphenicols									
Chloramphenicol	0│0	0│4	0│0	0│9	0│0	0│0	0│1	0(0%)	14(66.7%)
Cephalosporins (1st and 2nd gen.)									
Cefalotin	0│0	1│0	0│0	0│0	0│0	0│0	0│0	1(4.8%)	0(0%)
Cefoxitin	0│0	0│0	0│0	0│0	0│0	0│0	0│0	0 (0%)	0(0%)
Cefuroxime	0│0	0│0	0│0	3│0	0│0	0│0	0│0	3(14.3%)	0(0%)
Cefuroxime Axetil	0│0	0│0	0│0	3│0	0│0	0│0	0│0	3(14.3%)	0(0%)
Tetracyclines									
Minocycline	0│1	0│4	1│0	0│9	0│1	2│0	0│1	3(14.3%)	16(76.2%)
Tetracycline	0│1	0│4	0│0	0│9	0│1	0│0	1│0	1 (4.8%)	15(71.4%)
**Other**									
Penicillin & *β*-lactamase inhibitor									
Amoxicillin/Clavulanic Acid	0│0	0│0	0│0	1│0	0│0	0│0	0│0	1(4.8%)	0(0%)
Piperacillin/Tazobactam	0│0	0│0	0│0	0│0	0│0	0│0	0│0	0(0%)	0(0%)
Folate pathway inhibitors									
Trimethoprim	0│1	0│1	0│0	0│8	0│1	0│0	0│0	0(0%)	11(52.4%)
Folate pathway inhibitors/Sulfonamides									
Trimethoprim/Sulfamethoxazole	0│1	0│1	0│0	0│9	0│1	0│0	0│1	0(0%)	13(61.9%)
Nitrofurantoins									
Nitrofurantoin	0│0	0│0	0│0	0│0	0│0	0│0	0│0	0(0%)	0(0%)
ESBL (%)	–	–	–	–	–	–	–	–	0(0%)
MDR (%)	1(100%)	4(100%)	–	9(100%)	1(100%)	–	1(100%)	–	16(76.2%)

**Table C.2:** 

Class and antimicrobial	Group C (n = 37)											
	Albany	Bareilly	Bovismor-bificans	Braenderup	Infantis	Kentucky	Litchfield	Ohio	Potsdam	Rissen	Intermediate resistant (%)	Fully resistant (%)
No. isolates	1	1	3	13	3	1	3	2	1	9		
Samples (n = 30) (%)	1 (3.3%)	1 (3.3%)	1 (3.3%)	6 (20%)	1 (3.3%)	1 (3.3%)	3 (10%)	1 (3.3%)	1 (3.3%)	3 (10%)		
**Highest priority-critically important**												
Cephalosporins (3rd & 4th gen.)												
Cefepime	0│0	0│0	0│0	0│0	0│0	0│0	0│0	0│0	0│0	0│0	0(0%)	0(0%)
Cefixime	0│0	0│0	0│0	0│1	0│3	0│0	0│0	0│0	0│0	0│0	0(0%)	4(10.8%)
Cefotaxime	0│0	0│0	0│0	0│1	0│3	0│0	0│0	0│0	0│0	0│0	0(0%)	4(10.8%)
Ceftazidime	0│0	0│0	0│0	0│0	0│0	0│0	0│0	0│0	0│0	0│0	0(0%)	0(0%)
Ceftriazone	0│0	0│0	0│0	0│1	0│3	0│0	0│0	0│0	0│0	0│0	0(0%)	4(10.8%)
Polyymyxins												
Colistin	0│0	0│0	0│0	0│0	0│0	0│0	0│0	0│0	0│0	0│0	0 (0%)	0(0%)
Quinolones												
Ciprofloxacin	1│0	0│1	0│0	2│8	0│3	1│0	1│2	0│0	0│1	4│0	9(24.3%)	15(40.5%)
Levofloxacin	1│0	1│0	0│0	10│0	3│0	0│0	2│1	0│0	0│1	3│1	20(54.1%)	3(8.1%)
Moxifloxacin	0│0	0│0	0│0	1│0	0│0	0│0	1│1	0│0	0│1	0│0	2 (5.4%)	2(5.4%)
Nalidixic Acid	0│1	0│0	0│0	0│2	0│3	0│0	0│1	0│0	0│1	0│0	0(0%)	8(21.6%)
Ofloxacin	0│1	0│1	0│0	0│10	0│3	0│0	0│3	0│0	0│1	1│3	1(2.7%)	22 (59.5%)
**High priority-critically important**												
Aminoglycosides												
Amikacin	0│0	0│0	0│0	0│0	0│0	0│0	0│0	0│0	0│0	0│0	0(0%)	0 (0%)
Gentamicin	0│0	0│0	0│0	0│1	0│3	0│0	0│0	0│0	0│1	0│0	0(0%)	5(13.5%)
Tobramycin	0│0	1│0	0│0	0│1	0│3	0│0	1│0	0│0	1│0	0│0	3(8.1%)	4 (10.8%)
Carbapenems and other penems												
Ertapenem	0│0	0│0	0│0	0│0	0│0	0│0	0│0	0│0	0│0	0│0	0(0%)	0(0%)
Imipenem	0│0	0│0	0│0	0│0	0│0	0│0	0│0	0│0	0│0	0│0	0(0%)	0(0%)
Meropenem	0│0	0│0	0│0	0│0	0│0	0│0	0│0	0│0	0│0	0│0	0(0%)	0(0%)
Glycylcyclines												
Tigecycline	0│0	0│0	0│0	0│0	0│0	0│0	0│0	0│0	0│0	0│0	0(0%)	0 (0%)
Monobactams												
Aztreonam	0│0	0│0	0│0	0│1	0│3	0│0	0│0	0│0	0│0	0│0	0(0%)	4 (10.8%)
Penicillins												
Ampicillin	0│1	0│1	0│0	0│1	0│3	0│0	0│3	0│0	0│1	0│9	0(0%)	19(51.4%)
Piperacillin	0│1	0│1	0│0	0│1	0│3	0│0	0│3	0│0	0│1	0│9	0(0%)	19(51.4%)
Ticarcillin	0│1	0│1	0│0	0│1	0│3	0│0	0│3	0│0	0│1	0│9	0(0%)	19(51.4%)
**Highly important**												
Amphenicols												
Chloramphenicol	0│1	0│1	0│0	0│1	0│3	0│0	0│3	0│0	0│1	1│6	1(2.7%)	16(43.2%)
Cephalosporins (1st and 2nd gen.)												
Cefalotin	0│0	0│0	0│0	0│1	0│3	0│0	0│0	0│0	0│0	0│0	0(0%)	4(10.8%)
Cefoxitin	0│0	0│0	0│0	0│0	3│0	0│0	0│0	0│0	0│0	0│0	3(8.1%)	0(0%)
Cefuroxime	0│0	0│0	0│0	0│1	0│3	0│0	0│0	0│0	0│0	2│0	2(5.4%)	4(10.8%)
Cefuroxime Axetil	0│0	0│0	0│0	0│1	0│3	0│0	0│0	0│0	0│0	2│0	2(5.4%)	4(10.8%)
Tetracyclines												
Minocycline	0│0	0│0	0│0	0│1	0│3	0│0	0│3	0│0	0│1	0│9	0(0%)	17(45.9%)
Tetracycline	0│1	0│0	0│0	0│1	0│3	0│0	0│3	0│0	0│1	0│9	0(0%)	18(48.6%)
**Other**												
Penicillin & *β*-lactamase inhibitor												
Amoxicillin/Clavulanic Acid	1│0	0│0	0│0	0│0	0│0	0│0	0│0	0│0	0│0	0│0	1(2.7%)	0(0%)
Piperacillin/Tazobactam	0│0	0│0	0│0	0│0	0│0	0│0	0│0	0│0	0│0	0│0	0(0%)	0(0%)
Folate pathway inhibitors												
Trimethoprim	0│1	0│0	0│0	0│3	0│0	0│0	0│3	0│0	0│0	0│9	0(0%)	16(43.2%)
Folate pathway inhibitors/Sulfonamides												
Trimethoprim/Sulfamethoxazole	0│1	0│0	0│0	0│3	0│0	0│0	0│3	0│0	0│1	0│9	0(0%)	17(45.9%)
Nitrofurantoins												
Nitrofurantoin	0│0	0│0	0│0	6│1	0│3	0│0	0│0	0│0	0│0	0│0	6(16.2%)	4(10.8%)
ESBL (%)	–	–	–	1 (7.7%)	3 (100%)	–	–	–	–	–	–	4(10.8%)
MDR (%)	1(100%)	1 (100%)	–	1(7.7%)	3(100%)	–	3(100%)	–	1(100%)	9(100%)	–	19(51.4%)

**Table C.3:** 

Class and antimicrobial	Group E (n = 21)						
	Anatum	Give	London	Senftenberg	Weltevreden	Intermediate resistant (%)	Fully resistant (%)
No. isolates	11	4	1	3	2		
Samples (n = 30) (%)	5 (16.7%)	2 (6.7%)	1 (3.3%)	1 (3.3%)	1 (3.3%)		
**Highest priority-critically important**							
Cephalosporins (3rd & 4th gen.)							
Cefepime	0│0	0│0	0│0	0│0	0│0	0(0%)	0 (0%)
Cefixime	0│0	0│3	0│0	0│0	0│0	0(0%)	3(14.3%)
Cefotaxime	0│0	0│3	0│0	0│0	0│0	0(0%)	3(14.3%)
Ceftazidime	0│0	0│3	0│0	0│0	0│0	0(0%)	3(14.3%)
Ceftriazone	0│0	0│3	0│0	0│0	0│0	0(0%)	3(14.3%)
Polyymyxins							
Colistin	0│0	0│0	0│0	0│0	0│0	0 (0%)	0 (0%)
Quinolones							
Ciprofloxacin	2│0	0│4	0│0	0│0	0│0	2(9.5%)	4 (19%)
Levofloxacin	0│0	0│4	0│0	0│0	0│0	0(0%)	4 (19%)
Moxifloxacin	0│0	0│4	0│0	0│0	0│0	0(0%)	4 (19%)
Nalidixic Acid	0│0	0│4	0│0	0│0	0│0	0(0%)	4 (19%)
Ofloxacin	0│0	0│4	0│0	0│0	0│0	0(0%)	4 (19%)
**High priority-critically important**							
Aminoglycosides							
Amikacin	0│0	0│0	0│0	0│0	0│0	0(0%)	0 (0%)
Gentamicin	0│0	0│3	0│0	0│0	0│0	0(0%)	3(14.3%)
Tobramycin	0│0	0│3	0│0	0│0	0│0	0(0%)	3(14.3%)
Carbapenems and other penems							
Ertapenem	0│0	0│0	0│0	0│0	0│0	0(0%)	0(0%)
Imipenem	0│0	0│0	0│0	0│0	0│0	0(0%)	0(0%)
Meropenem	0│0	0│0	0│0	0│0	0│0	0(0%)	0(0%)
Glycylcyclines							
Tigecycline	0│0	0│3	0│0	0│0	0│0	0(0%)	3(14.3%)
Monobactams							
Aztreonam	0│0	0│3	0│0	0│0	0│0	0(0%)	3(14.3%)
Penicillins							
Ampicillin	0│7	0│4	0│1	0│0	0│0	0(0%)	12(57.1%)
Piperacillin	0│7	0│4	0│1	0│0	0│0	0(0%)	12(57.1%)
Ticarcillin	0│7	0│4	0│1	0│0	0│0	0(0%)	12(57.1%)
**Highly important**							
Amphenicols							
Chloramphenicol	0│3	0│4	0│1	0│0	0│0	0(0%)	8(38.1%)
Cephalosporins (1st and 2nd gen.)							
Cefalotin	0│0	0│3	0│0	0│0	0│0	0 (0%)	3(14.3%)
Cefoxitin	0│0	0│0	0│0	0│0	0│0	0 (0%)	0(0%)
Cefuroxime	5│0	0│3	0│0	0│0	0│0	5(23.8%)	3(14.3%)
Cefuroxime Axetil	11│0	0│3	0│0	0│0	0│0	11(52.4%)	3(14.3%)
Tetracyclines							
Minocycline	3│3	0│4	0│1	0│0	0│0	3(14.3%)	8(38.1%)
Tetracycline	0│10	0│4	0│1	0│0	0│0	0(0%)	15(71.4%)
**Other**							
Penicillin & *β*-lactamase inhibitor							
Amoxicillin/Clavulanic Acid	0│0	0│0	0│0	0│0	0│0	0(0%)	0(0%)
Piperacillin/Tazobactam	0│0	0│0	0│0	0│0	0│0	0(0%)	0(0%)
Folate pathway inhibitors							
Trimethoprim	0│9	0│4	0│1	0│0	0│0	0(0%)	14(66.7%)
Folate pathway inhibitors/Sulfonamides							
Trimethoprim/Sulfamethoxazole	0│3	0│4	0│1	0│0	0│0	0(0%)	8(38.1%)
Nitrofurantoins							
Nitrofurantoin	0│0	0│0	0│0	0│0	0│0	0(0%)	0 (0%)
ESBL (%)		3 (75%)	–	–	–	–	3(14.3%)
MDR (%)	10(90.9%)	4(100%)	1(100%)	–	–	–	15(71.4%)

**Table C.4:** 

Class and antimicrobial	Other serogroups (N = 11)					
	Enteritidis	Kedougou	Poona	Urbana	Intermediate resistant (%)	Fully resistant (%)
No. isolates	4	2	3	2		
Samples (n = 30) (%)	2 (6.7%)	1 (3.3%)	1 (3.3%)	1 (3.3%)		
**Highest priority-critically important**						
Cephalosporins (3rd & 4th gen.)						
Cefepime	0│0	0│0	0│0	0│0	0(0%)	0(0%)
Cefixime	0│0	0│0	0│0	0│0	0(0%)	0(0%)
Cefotaxime	0│0	0│0	0│0	0│0	0(0%)	0(0%)
Ceftazidime	0│0	0│0	0│0	0│0	0(0%)	0(0%)
Ceftriazone	0│0	0│0	0│0	0│0	0(0%)	0(0%)
Polyymyxins						
Colistin	0│1	0│0	0│0	0│0	0(0%)	1(9.1%)
Quinolones						
Ciprofloxacin	3│0	1│0	0│0	0│0	4(36.4%)	0 (0%)
Levofloxacin	3│0	1│0	0│0	0│0	4(36.4%)	0 (0%)
Moxifloxacin	0│0	0│0	0│0	0│0	0 (0%)	0 (0%)
Nalidixic Acid	0│3	0│0	0│0	0│0	0 (0%)	3 (27.3%)
Ofloxacin	3│0	0│1	0│0	0│0	3 (27.3%)	1 (9.1%)
**High priority-critically important**						
Aminoglycosides						
Amikacin	0│0	0│0	0│0	0│0	0(0%)	0(0%)
Gentamicin	0│0	0│0	0│0	0│0	0(0%)	0(0%)
Tobramycin	0│0	0│0	0│0	0│0	0(0%)	0(0%)
Carbapenems and other penems						
Ertapenem	0│0	0│0	0│0	0│0	0(0%)	0(0%)
Imipenem	0│0	0│0	0│0	0│0	0(0%)	0(0%)
Meropenem	0│0	0│0	0│0	0│0	0(0%)	0(0%)
Glycylcyclines						
Tigecycline	0│0	0│0	0│0	0│0	0 (0%)	0(0%)
Monobactams						
Aztreonam	0│0	0│0	0│0	0│0	0 (0%)	0(0%)
Penicillins						
Ampicillin	0│3	0│0	0│0	0│0	0(0%)	3(27.3%)
Piperacillin	0│3	0│0	0│0	0│0	0(0%)	3(27.3%)
Ticarcillin	0│3	0│0	0│0	0│0	0(0%)	3(27.3%)
**Highly important**						
Amphenicols						
Chloramphenicol	0│0	0│0	0│0	0│0	0(0%)	0 (0%)
Cephalosporins (1st and 2nd gen.)						
Cefalotin	2│0	0│0	0│0	0│0	2(18.2%)	0(0%)
Cefoxitin	0│0	0│0	0│0	0│0	0(0%)	0(0%)
Cefuroxime	0│0	1│0	0│0	0│0	1 (9.1%)	0(0%)
Cefuroxime Axetil	2│0	1│0	0│0	0│0	3(27.3%)	0(0%)
Tetracyclines						
Minocycline	0│3	1│0	0│0	0│0	1(9.1%)	3(27.3%)
Tetracycline	0│3	0│0	0│0	0│0	0(0%)	3(27.3%)
**Other**						
Penicillin & *β*-lactamase inhibitor						
Amoxicillin/Clavulanic Acid	1│0	0│0	0│0	0│0	1(9.1%)	0(0%)
Piperacillin/Tazobactam	0│0	0│0	0│0	0│0	0(0%)	0(0%)
Folate pathway inhibitors						
Trimethoprim	0│0	0│0	0│0	0│0	0(0%)	0(0%)
Folate pathway inhibitors/Sulfonamides						
Trimethoprim/Sulfamethoxazole	0│0	0│0	0│0	0│0	0(0%)	0(0%)
Nitrofurantoins						
Nitrofurantoin	0│3	0│0	0│0	0│0	0(0%)	3(27.3%)
ESBL (%)	–	–	–	–	–	0(0%)
MDR (%)	3(75%)	–	–	–	–	3(27.3%)

**Table C.5:** 

Class and antimicrobial Isolates (n = 90)	All serovars			CLSI 2017 breakpoints	
	No. isolates (N = 90)	Intermediate resistant (%)	Fully resistant (%)		
				Sensitive	Resistant
**Highest priority-critically important**					
Cephalosporins (3rd & 4th gen.)					
Cefepime	0│0	0(0%)	0(0%)	≤2	≥16
Cefixime	0│7	0(0%)	7(7.8%)	≤1	≥4
Cefotaxime	0│7	0(0%)	7(7.8%)	≤1	≥4
Ceftazidime	0│3	0(0%)	3(3.3%)	≤4	≥16
Ceftriazone	0│7	0(0%)	7(7.8%)	≤1	≥4
Polyymyxins					
Colistin	0│1	0 (0%)	1(1.1%)	≤2	>2
Quinolones					
Ciprofloxacin	23│30	23(25.6%)	30(33.3%)	≤0.06	≥1
Levofloxacin	33│14	33(36.7%)	14(15.6%)	≤0.12	≥2
Moxifloxacin	3│13	3(3.3%)	13(14.4%)	≤2	≥8
Nalidixic Acid	0│22	0(0%)	22(24.4%)	≤16	≥32
Ofloxacin	4│43	4(4.4%)	43(47.8%)	≤0.12	≥2
**High priority-critically important**					
Aminoglycosides					
Amikacin	0│0	0(0%)	0(0%)	≤16	≥64
Gentamicin	0│15	0(0%)	15(16.7%)	≤4	≥16
Tobramycin	10│7	10(11.1%)	7(7.8%)	≤4	≥16
Carbapenems and other penems					
Ertapenem	0│0	0(0%)	0(0%)	≤0.5	≥2
Imipenem	0│0	0(0%)	0(0%)	≤1	≥4
Meropenem	0│0	0(0%)	0(0%)	≤1	≥4
Glycylcyclines					
Tigecycline	3│3	3(3.3%)	3(3.3%)	≤2	≥8
Monobactams					
Aztreonam	0│7	0(0%)	7(7.8%)	≤4	≥16
Penicillins					
Ampicillin	0│50	0(0%)	50(55.6%)	≤8	≥32
Piperacillin	0│50	0(0%)	50(55.6%)	≤16	≥128
Ticarcillin	1│49	1(1.1%)	49(54.4%)	≤16	≥128
**Highly important**					
Amphenicols					
Chloramphenicol	1│38	1 (1.1%)	38 (42.2%)	≤8	≥32
Cephalosporins (1st and 2nd gen.)					
Cefalotin	3│7	3(3.3%)	7(7.8%)	≤8	≥32
Cefoxitin	3│0	3(3.3%)	0 (0%)	≤8	≥32
Cefuroxime	11│7	11(12.2%)	7(7.8%)	≤8	≥32
Cefuroxime Axetil	19│7	19(21.1%)	7(7.8%)	≤4	≥32
Tetracyclines					
Minocycline	7│44	7(7.8%)	44(48.9%)	≤4	≥16
Tetracycline	1│51	1(1.1%)	51(56.7%)	≤4	≥16
**Other**					
Penicillin & *β*-lactamase inhibitor					
Amoxicillin/Clavulanic Acid	3│0	3(3.3%)	0(0%)	≤8/4	≥32/16
Piperacillin/Tazobactam	0│0	0(0%)	0(0%)	≤16/4	≥128/4
Folate pathway inhibitors					
Trimethoprim	0│41	0(0%)	41(45.6%)	≤8	≥16
Folate pathway inhibitors/Sulfonamides					
Trimethoprim/Sulfamethoxazole	0│38	0(0%)	38(42.2%)	≤2/38	≥4/76
Nitrofurantoins					
Nitrofurantoin	6│7	6(6.7%)	7 (7.8%)	≤32	≥128
ESBL (%)	–	–	7(7.8%)	–	–
MDR (%)	–	–	53(58.9%)	–	–

**Table D:** Phenotypic antimicrobial susceptibility results for Vibrio spp. isolates (n = 133). The figures correspond to the number of intermediate resistant, followed by the number of fully resistant isolates.

Class and antimicrobial	No. isolates and percent resistant (%) (n = 133 isolates)								Disk	Zone diameter (mm)	
	*V. parahaemolyticus* (n = 64)	*V. navarrensis* (n = 23)	*V. alginolyticus* (n = 21)	*V. cholerae* non-O1 (n = 15)	*V. vulnificus* (n = 6)	*V. fluvialis* (n = 4)	Total intermediate resistant (%)	Total fully resistant (%)	antimicrobial		
									(*μ*g/mL)	Sensitive	Resistant
**Highest priority-critically important**											
*Cephalosporins (3*rd *&4*th *gen.)*											
Cefotaxime	3ǀ20	10ǀ2	0ǀ0	8ǀ0	2ǀ0	0ǀ0	23 (17.3%)	22 (16.5%)	30	≥23	≤14
Ceftazidime	3ǀ10	0ǀ0	0ǀ0	1ǀ1	0ǀ0	0ǀ0	4 (3.0%)	11 (8.3%)	30	≥18	≤14
*Quinolones*											
Ciprofloxacin	9ǀ7	0ǀ0	1ǀ0	2ǀ0	1ǀ0	0ǀ0	13 (9.8%)	7 (5.3%)	5	≥21	≤15
Ofloxacin	3ǀ6	0ǀ0	0ǀ0	2ǀ0	0ǀ0	0ǀ0	5 (3.8%)	6 (4.5%)	5	≥16	≤12
**High priority-critically important**											
*Aminoglycosides*											
Amikacin	29ǀ1	1ǀ0	1ǀ0	0ǀ0	1ǀ0	0ǀ0	32 (24.0%)	1 (0.8%)	30	≥17	≤14
Gentamicin	7ǀ0	1ǀ0	1ǀ0	0ǀ0	0ǀ0	0ǀ0	9 (6.8%)	0 (0%)	10	≥15	≤12
*Carbapenems and other penems*											
Imipenem	0ǀ2	0ǀ1	0ǀ0	1ǀ0	0ǀ0	0ǀ0	1 (0.8%)	3 (2.3%)	10	≥16	≤13
*Penicillins*											
Ampicillin	0ǀ64	6ǀ12	0ǀ21	3ǀ8	1ǀ2	0ǀ3	10 (7.5%)	110 (82.7%)	10	≥17	≤13
Amoxicillin-clavulanic acid	1ǀ6	8ǀ4	0ǀ0	3ǀ5	2ǀ0	3ǀ0	17 (12.8%)	15 (11.3%)	20ǀ10	≥18	≤13
**Highly important**											
*Amphenicols*											
Chloramphenicol	3ǀ1	1ǀ0	0ǀ0	1ǀ0	1ǀ0	0ǀ0	6 (4.5%)	1 (0.8%)	30	≥18	≤12
*Tetracyclines*											
Tetracycline	15ǀ7	3ǀ1	0ǀ0	1ǀ1	0ǀ1	0ǀ0	19 (14.3%)	10 (7.5%)	30	≥19	≤14
*Folate pathway inhibitors & sulfonamides*											
Trimethoprim/sulfamethoxazole	7ǀ17	3ǀ3	1ǀ0	0ǀ3	1ǀ2	1ǀ0	13 (9.8%)	25 (18.8%)	1.25/23.75	≥16	≤10
**ESBL**	18	0	0	0	0	0	–	18 (13.5%)			
**MDR**	18	0	0	0	0	0	–	18 (13.5%)			
